# “Just One More Rep!” – Ability to Predict Proximity to Task Failure in Resistance Trained Persons

**DOI:** 10.3389/fpsyg.2020.565416

**Published:** 2020-12-23

**Authors:** Cedrik Armes, Henry Standish-Hunt, Patroklos Androulakis-Korakakis, Nick Michalopoulos, Tsvetelina Georgieva, Alex Hammond, James P. Fisher, Paulo Gentil, Jürgen Giessing, James Steele

**Affiliations:** ^1^Centre for Health, Exercise and Sport Science, School of Sport, Health and Social Sciences, Solent University, Southampton, United Kingdom; ^2^Department of Human Movement Sciences, Vrije Universiteit Amsterdam, Amsterdam, Netherlands; ^3^Department of Physics, University of Patras, Patras, Greece; ^4^Faculty of Physical Education and Dance, Federal University of Goiás, Goiânia, Brazil; ^5^Institute for Sport Science, University of Koblenz and Landau, Landau, Germany; ^6^ukactive Research Institute, ukactive, London, United Kingdom

**Keywords:** perception, effort, physical, strength, exercise, performance

## Abstract

In resistance training, the use of predicting proximity to momentary task failure (MF, i.e., maximum effort), and repetitions in reserve scales specifically, is a growing approach to monitoring and controlling effort. However, its validity is reliant upon accuracy in the ability to predict MF which may be affected by congruence of the perception of effort compared with the actual effort required. The present study examined participants with at least 1 year of resistance training experience predicting their proximity to MF in two different experiments using a deception design. Within each experiment participants performed four trials of knee extensions with single sets (i.e., bouts of repetitions) to their self-determined repetition maximum (sdRM; when they predicted they could not complete the next repetition if attempted and thus would reach MF if they did) and MF (i.e., where despite attempting to do so they could not complete the current repetition). For the first experiment (*n* = 14) participants used loads equal to 70% of a one repetition maximum (1RM; i.e., the heaviest load that could be lifted for a single repetition) performed in a separate baseline session. Aiming to minimize participants between day variability in repetition performances, in the second separate experiment (*n* = 24) they used loads equal to 70% of their daily isometric maximum voluntary contraction (MVC). Results suggested that participants typically under predicted the number of repetitions they could perform to MF with a meta-analytic estimate across experiments of 2.0 [95%CIs 0.0 to 4.0]. Participants with at least 1 year of resistance training experience are likely not adequately accurate at gauging effort in submaximal conditions. This suggests that perceptions of effort during resistance training task performance may not be congruent with the actual effort required. This has implications for controlling, programming, and manipulating the actual effort in resistance training and potentially on the magnitude of desired adaptations such as improvements in muscular hypertrophy and strength.

## Introduction

Prolonged performance of physical tasks with fixed absolute demands results in a reduction in the capacity to meet their demands (i.e., fatigue), and thus a requirement for greater effort to maintain performance. As a result of this, the perception of that effort also increases (Horstman et al., 1979; Noakes, 2004). This appears to be the case over varying exercise modalities including both endurance and resistance training (Horstman et al., 1979; Pincivero et al., 2004; Marcora and Staiano, 2010). Though rating of perceived effort (RPE) scales are widely employed in physical tasks, scales have been developed that are aimed at utilizing the feedback from increasing perceptions of fatigue and effort in order to predict proximity to task failure (Coquart et al., 2012; Helms et al., 2016). The application of predictions of proximity to task failure has been a particularly popular approach within resistance training in recent years to manipulate and control the intensity of effort employed in a given bout (Hackett et al., 2012, 2016; Helms et al., 2016; Zourdos et al., 2016).

Within physical tasks such as resistance exercise the intensity of effort employed has been defined as the task demands (i.e., the load) relative to the current ability to meet those demands (i.e., a person’s strength; Steele, 2014, 2020; Steele et al., 2017b, 2019). Considering this, *maximal* effort is anchored at the set endpoint where the participant reaches momentary task failure (MF, i.e., where despite attempting to do so the trainee cannot complete the current repetition; Steele, 2014; Steele et al., 2017b). MF has also been argued to be the most appropriate way to control for effort intra- and inter-individually (Dankel et al., 2016). However, to better understand applications of *submaximal* intensities of effort (i.e., set end-points that occur at different proximities to MF) ‘repetitions in reserve’ (RIR) scales have been developed and employed (Hackett et al., 2012, 2016; Helms et al., 2016; Zourdos et al., 2016). RIR scales assess or control effort by participants estimating how many repetitions they can perform before reaching MF. These scales have been argued to be a more valid method of representing effort during resistance training when compared to traditional RPE scales or the use of relative demands from a prior test of strength (i.e., % of one repetition maximum [1RM]; Hackett et al., 2012; Helms et al., 2016; Steele et al., 2017a). Indeed, traditional RPE scales often result in submaximal ratings even at MF (Steele et al., 2017c). Further, the numbers of possible repetitions prior to MF at the same relative loads (%1RM) vary between exercises and individuals (Steele, 2014; Steele et al., 2017a,b). Thus, RIR scales might provide a more accurate way of controlling for effort during resistance training. Further, predictive ability offers a behavioral test of the congruence of perception of effort and actual effort in resistance exercise tasks.

An assumption inherent in use of RIR scales to provide valid control of intensity of effort is that participants can accurately predict their number of repetitions until MF. Several recent studies have examined this predictive ability under a variety of conditions, including *a priori* to beginning the exercise (Steele et al., 2017a; Emanuel et al., 2020), and at varying proximities to MF during the exercise (Hackett et al., 2012, 2016; Altoé Lemos et al., 2017; Zourdos et al., 2019; Hughes et al., 2020; Mansfield et al., 2020). Most have shown that people are inaccurate in their predictions suggesting that, when using an RIR based prescription, they may be training at a lower actual effort than intended. This may have implications for training outcomes from interventions. A recent meta-analysis reported little difference between training to MF, or not (Grgic et al., 2020). However, some studies comparing groups training to MF and those who stopped at a self-determined repetition maximum (sdRM, i.e., when a person predicts they could not complete the next repetition if attempted and thus would reach MF if they did; Steele et al., 2017b) have shown greater responses when training to MF (Giessing et al., 2016a,b). This may be due to participants stopping further from MF than intended due to their poor ability to predict actual proximity to MF.

Throughout a bout of resistance exercise, the combined perceptions associated with that gestalt experience (i.e., perceived fatigue, effort, and discomfort) typically intensify with closer proximity to MF. Thus, we might expect the accuracy of prediction should increase the closer to MF a person is when they make it. Indeed, prediction has been shown to be more accurate when using heavier loads (i.e., where fewer repetitions are possible such that any given repetition is closer to MF; Altoé Lemos et al., 2017; Steele et al., 2017a). Further, accuracy increases with subsequent sets possibly due to practice, or lingering fatigue (Hackett et al., 2012; Emanuel et al., 2020; Mansfield et al., 2020). However, only one study has examined varying proximities to failure (Zourdos et al., 2019). Zourdos et al. (2019) examined the validity of predictions of 5RIR, 3RIR, and 1RIR (i.e., 5, 3, and 1 repetition in reserve). They found that accuracy improved with proximity to MF, but participants were still inaccurate even for 1RIR. Further, these were previously trained individuals. Indeed, it has been argued that RIR might be best applied in trained persons (Helms et al., 2016). Although, there is some contrasting evidence regarding the effect of prior experience on accuracy of prediction (Hackett et al., 2016; Steele et al., 2017a). Considering previous findings and the interest in quantifying effort through RIR scales, there is a need to examine this further. Indeed, given the increasing predictive accuracy with increasing proximity to MF, we might expect predictive ability to be at its greatest when participants are attempting to get as close to, but not reach, MF. The use of RIR implies *complete* repetitions that a person predicts they can perform. As such, 1RIR would mean that a person estimates they could perform one more complete repetition. Contrastingly, a 0RIR would mean they estimate that they would reach MF on the subsequent repetition (Helms, Personal Communication). No prior research has examined predictive ability for a 0RIR, or what Steele et al. (2017b) have referred to as the sdRM. Therefore, the aim of this study was to examine ability to predict proximity to MF at the sdRM/0RIR. In two separate experiments using a deception design, participants experienced in resistance training (>1 year) were tested over four trials whilst performing one set of knee extensions to either MF or sdRM.

## Materials and Methods

### Experimental Approach

The study was approved by the Health, Exercise, and Sport Science ethics committee at Solent University (ID: standish-hunt2018). There were two separate experiments conducted in this study for which separate samples of participants were recruited. Testing procedures involved performing knee extensions on a knee extension dynamometer (MedX, Ocala, FL, United States; Experiment 1 and 2) or a knee extension resistance machine (Cybex, Medway, MA, United States; Experiment 1). In both experiments, participants underwent four resistance exercise trials involving single sets (i.e., bouts of repetitions) of knee extensions with at least 48 h in between to determine their ability to accurately identify their sdRM (i.e., 0RIR). Two of the resistance exercise trials were comprised of one set until their sdRM and the other two trials of one set until MF in a randomized order. To reduce demand characteristics (where participants’ expectations of the experiments purpose might influence their performance) from invalidating the results, a deception was used blinding the participants to the actual goal of the study. Participants were informed that this was a reliability study examining similarities within the repeated identical condition trials (i.e., the reliability of sdRM or MF repetition performance between days). However, the study actually investigated the agreement between the different conditions. This was aimed at addressing participants consciously or unconsciously adapting their behavior, such that their apparent predictive ability was influenced (i.e., adjusting the number of repetitions performed in either condition to make it appear as though predictive accuracy was greater). In debrief after completion of the experiments, participants were asked whether they knew what the purpose of the study was to which all confirmed that they thought it was a reliability study as they were informed. Thus, it was confirmed that no participants had determined the true purpose of the study suggesting the deception had been successful.

### Participants

Originally 11 participants were recruited for Experiment 1. From the initial data collected in Experiment 1 we produced an exploratory linear mixed model using the ‘lme4’ package (Bates et al., 2015) in R (version 3.6.1; R Core Team, 2020) to examine the fixed effect of condition adjusted for the fixed effect of day and allowing random intercepts by participant. Then, using the ‘simr’ package (Green and MacLeod, 2015), this model was extended to 100 participants and a simulation (1000 resamples) conducted to allow power curve analysis to be performed (see Supplementary Materials). Simulation showed that, for >80% power, ∼30 participants would be required at an alpha level of 0.05 and ∼25 participants at an alpha level of 0.1. As such, we aimed to recruit ∼30 for Experiment 2 to be able to exclude a zero effect. However, we were unable to achieve the intended 30 participants due to cessation of data collection as a result of ‘lockdown’ measures because of COVID-19. Hence, the final sample for Experiment 2 was 24 participants. An opportunity to collect additional data for Experiment 1 in another location and using a knee extension resistance machine (Cybex, Medway, MA, United States)^1^ resulted in a final sample of 14 participants, but was also cut short due to the same reasons. Thus, the results of either experiment should be treated with caution individually. To somewhat overcome the sample issues, we conducted an internal meta-analysis (see below).

The final samples were *n* = 14 (11 males aged 22 ± 2 years and 3 females aged 20 ± 1 years) for Experiment 1, and *n* = 24 (20 male aged 27 ± 6 years and 4 females aged 24 ± 2 years) for Experiment 2. None of the participants took part in both experiments. Participants were required to have a resistance training experience of at least 1 year and to have abstained from any strenuous physical activity for 72-h prior to testing. All participants were provided with a participant information sheet including the deceptive purpose of the study and gave written informed consent. The participants had to complete a physical activity readiness questionnaire which covered any areas whereby there may be contraindications to the exercise (e.g., injury etc.). Participants were given the opportunity to withdraw from the study at any time and were debriefed after completion of the study.

### Experiment 1: Resistance Exercise Trials Based on Baseline 70%1RM

The testing procedure of Experiment 1 involved one baseline 1RM test and four resistance exercise trials (2x sdRM; 2x MF) where one set of knee extension resistance exercise for each condition was performed. All conditions were performed in a randomized order and separated by at least 48 h. Within the baseline session, participants’ range of motion (ROM) was determined by measuring their maximum knee extension and flexion angles. Following a warm-up using 50% of their estimated 1RM load, their 1RM was determined within a maximum of five attempts with 4-min rest between attempts. For some participants it was possible for the maximum resistance on the weight stack to be lifted for multiple repetitions and so 1RM was predicted using the Brzycki (1993) equation [predicted 1RM = load lifted/(1.0278 − (0.0278 × number of repetitions)] which has been shown to have a very high correlation to actual 1RM (*r* = 0.99; Nascimento et al., 2007). The load for the following four trials was calculated as 70% of their baseline 1RM. Subsequently, two sessions of submaximal sets to sdRM and two sessions of maximal sets to MF were performed.

Each session started with a warm-up involving one set of knee extensions at 50% of the calculated condition load with 8–10 repetitions, followed by a rest of 5 min after which the condition was performed. The previously determined ROM was set such that a ‘beep’ sound was provided by the dynamometer when at full extension/flexion to ensure that a full ROM was used for each repetition. Participants were instructed as follows. For the sdRM conditions they were instructed to, immediately upon completing a given repetition, consider whether they felt they could complete the next if attempted; if they did not think they could complete another if attempted they were to stop there and inform the investigator. For the MF conditions they were instructed to, immediately upon completing a given repetition, always attempt the next repetition; this was to continue until they reached a point where despite their maximal effort they could not complete the concentric portion of a repetition. The total number of completed repetitions were examined for each condition (i.e., the repetition chosen to stop on during sdRM, and the last complete repetition prior to MF). Participants were encouraged to think carefully about their sdRM prediction during that condition and push as close to, but not actually reach MF, and to perform with maximal effort for the MF condition.

### Experiment 2: Resistance Exercise Trials Based on Daily 70%MVC

The testing procedure of Experiment 2 was the same as that used for Experiment 1 with one difference. We found that participants’ repetition performances between the trials but within conditions were highly variable in Experiment 1, potentially attributed to individual day-to-day variabilities in preparedness (e.g., fatigue, mental state, stress, prior sleep, muscle glycogen concentrations etc.). Hence in Experiment 2, we opted to perform a daily maximal voluntary contraction (MVC) to examine participants’ ‘daily max performance’ and allow us to normalize loads to each participants strength on the day of each resistance exercise trial. We chose MVCs as opposed to daily 1RMs, due to their brief nature and the minimal impact of fatigue that might affect the subsequent trial (Kennedy et al., 2015).

At the beginning of each session, following a warm-up and a practice isometric trial, participants performed an isometric MVC at 78° of flexion (previous testing in our lab suggests that most participants reach a peak torque at this angle) to determine their maximum voluntary torque in N⋅m. The load for each condition was thus calculated by 70% of their MVC in N⋅m for that day. The process of measuring MVCs was repeated before each session. Loads on the weight stack for the MedX Knee Extension are expressed in N⋅m and so we were able to normalize load against the MVC expressed in the same units. After a warm-up of 8–10 repetitions at 50% of their condition load followed by a rest of 5 min, the condition for that day was performed (i.e., sdRM or MF).

### Statistical Analysis

The dependent variable was the number of complete repetitions performed and the independent variable was the condition (sdRM and MF). Linear mixed modeling using Restricted Maximum Likelihood Estimation was used for analysis. Condition was modeled as a fixed factor with random intercepts by participants included. As each condition was performed across two sessions (days), each participant had two pairs of sdRM:MF repetitions. Thus, day was also adjusted for in the model as a fixed factor. Estimated marginal means with 95% confidence intervals (CI) were produced using the “emmeans” package. Contrasts were produced using both 95% and 90% CIs to support inferences regarding equivalence. Equivalence bands were determined based upon the between day reliability of repetitions performed to MF within each study based upon the half-width of the minimal detectable change (MDC), sometimes referred to as the minimal difference, as typically suggested for examination of equivalence (Lesaffre, 2008). The MDC was calculated for the two repeated MF trials as:

M⁢D⁢C=S⁢E⁢M⁢x⁢ 1.96⁢x⁢2

Where,

$$S\,E\,M=S\,D\,d/\sqrt{2}$$

And the SDd is the standard deviation of the difference scores between the two trials (Weir, 2005).

Lastly, we combined the results from the two Experiments using an internal meta-analysis to obtain an overall effect estimate (Goh et al., 2016). The ‘metafor’ (Viechtbauer, 2010) package was used to perform a random effects meta-analysis weighted by sample size to produce effect estimates using both 95% and 90% CIs.

Inferences were drawn primarily regarding the magnitude and uncertainty of each outcome, whether it be close to zero or the equivalence bands. We opted to avoid dichotomizing the existence of an effect and therefore did not employ traditional null hypothesis significance testing, which has been extensively discussed (Amrhein et al., 2019; McShane et al., 2019). Instead, we consider the implications of all results compatible with these data, from the lower limit to the upper limit of the CIs, with the greatest interpretive emphasis placed on the point estimate. All effect estimates are reported in their raw units (number of repetitions) to facilitate practical interpretation.

## Results

### Experiment 1: Resistance Exercise Trials Based on Baseline 70%1RM

**FIGURE 1:**
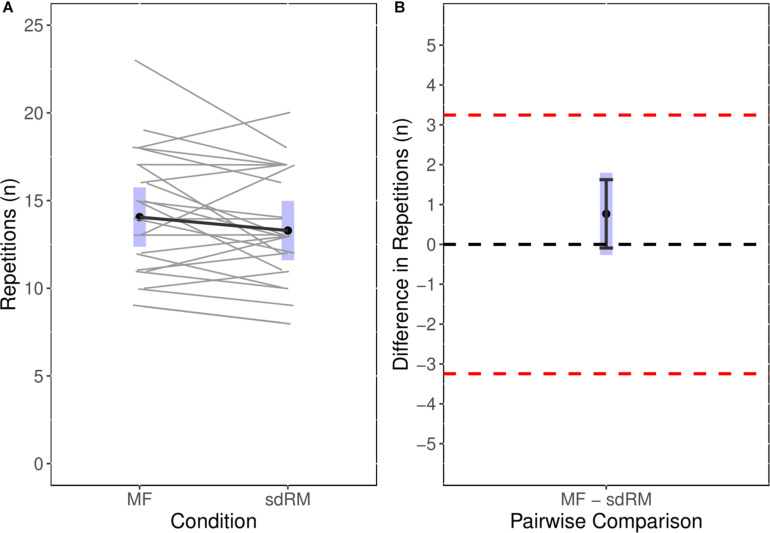
Experiment 1: **(A)** Estimated marginal means with individual paired data for number of repetitions performed in MF and sdRM; **(B)** estimated marginal mean for the pairwise comparison between MF and sdRM with both 95%CIs (*gray* band) and 90%CIs (black error bars) with the equivalence bands (dashed red line). Individual data are presented as paired observations within days (i.e., sdRM day 1 was paired with MF day 1) as this was adjusted for within the model. MF, momentary failure; sdRM, self-determined repetition maximum.

The point estimate for the number of repetitions performed during the sdRM condition was 13.3 with the 95%CIs suggesting compatibility with a range of 11.6 to 15.0 repetitions. For the MF condition the point estimate was 14.1 repetitions with the 95%CIs suggesting compatibility with a range of 12.4 to 15.8 repetitions. The paired contrast showed that the number of repetitions performed during the MF condition was 0.8 greater than during the sdRM condition. The 95%CIs ranged −0.26 to 1.8 and thus did not exclude a possible effect estimate of zero, though included possible estimates of as high as 1.8 repetitions. The 90%CIs ranged from −0.1 to 1.6. Notably, considering the MDC for Experiment 1 (3.2 repetitions), neither the point estimate nor 95% or 90% estimate intervals excluded its upper bound thus suggesting equivalence within the range of the MDC between the repetitions performed in both conditions. Figure 1 shows the individual paired comparisons (Session:Participant) across the conditions in addition to the paired contrast with both 95%CIs (gray band) and 90%CIs (black error bars) with the equivalence bands (dashed red line).

### Experiment 2: Resistance Exercise Trials Based on Daily 70%MVC

**FIGURE 2:**
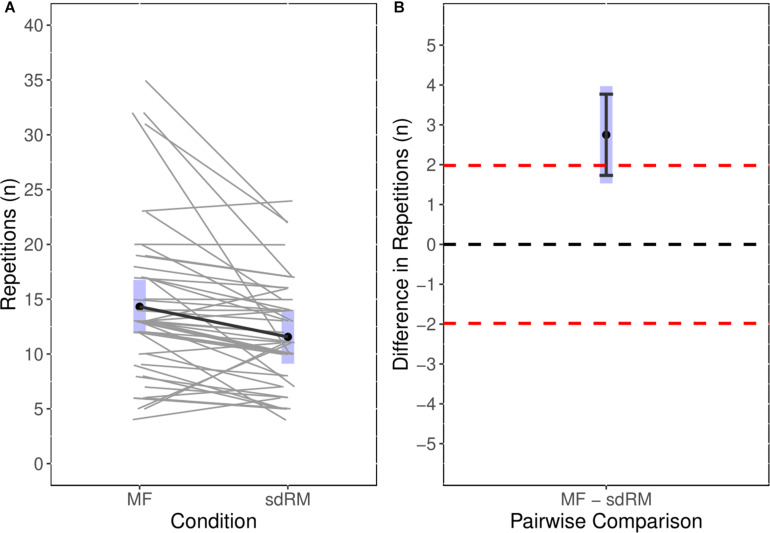
Experiment 2: **(A)** Estimated marginal means with individual paired data for number of repetitions performed in MF and sdRM; **(B)** estimated marginal mean for the pairwise comparison between MF and sdRM with both 95%CIs (*gray* band) and 90%CIs (black error bars) with the equivalence bands (dashed red line). Individual data are presented as paired observations within days (i.e., sdRM day 1 was paired with MF day 1) as this was adjusted for within the model. MF, momentary failure; sdRM, self-determined repetition maximum.

The point estimate for the number of repetitions performed during the sdRM condition was 11.6 with the 95%CIs suggesting compatibility with a range of 9.1 to 14.0 repetitions. For the MF condition the point estimate was 14.3 repetitions with the 95%CIs suggesting compatibility with a range of 11.9 to 16.8 repetitions. The paired contrast showed that the number of repetitions performed during the MF condition was 2.8 greater than during the sdRM condition. The 95%CIs ranged 1.5 to 4.0 and thus excluded a possible effect estimate of zero. The 90%CIs ranged from 1.7 to 3.8. Notably, considering the MDC for Experiment 1 (2.0 repetitions), the point estimate exceeded this; however, neither the 95% or 90% estimate intervals excluded its upper bound thus equivalence within the range of the MDC remains a possible compatible effect between the repetitions performed in both conditions. Figure 2 shows the individual paired comparisons (Session:Participant) across the conditions in addition to the paired contrast with both 95%CIs (gray band) and 90%CIs (black error bars) with the equivalence bands (dashed red line).

### Internal Meta-Analysis

**FIGURE 3:**
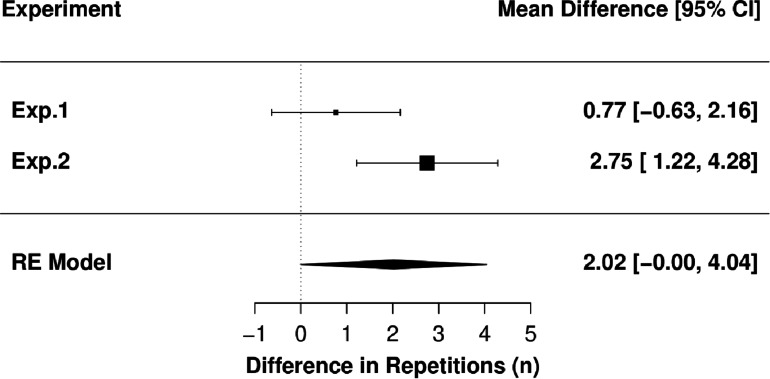
Forest plot of both experiments with 95%CIs; RE, random-effects.

**FIGURE 4:**
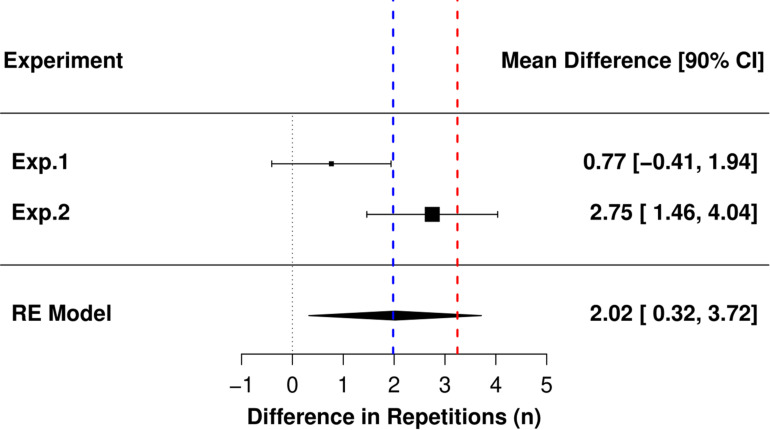
Forest plot of both experiments with 90%CIs in addition to the upper equivalence bands from both Experiment 1 (dashed red line) and Experiment 2 (dashed blue line); RE, random-effects.

The paired contrast estimate from the random effects meta-analysis showed that the number of repetitions performed during the MF condition was 2.0 greater than during the sdRM condition. The 95%CIs ranged 0.0 to 4.0 and thus just included a possible effect estimate of zero. The 90%CIs ranged from 0.3 to 3.7. Figure 3 presents the forest plot with 95%CIs and Figure 4 presents the forest plot with 90%CIs in addition to the upper equivalence bands from both Experiment 1 (dashed red line) and Experiment 2 (dashed blue line).

## Discussion

The results of the present study suggest on average participants under predicted the number of repetitions they could perform to MF. Compared to the actual number of complete repetitions in sets to MF, the number of complete repetitions in the sdRM condition were typically lower. However, in Experiment 1 this did not exceed the MDC. Thus, based upon the between day variability in repetition performance, the repetition numbers were inferred to be equivalent between conditions. For Experiment 2, as expected, there was a reduction in the between day variability as seen by the reduced MDC; indeed the intraclass correlation coefficient [3,1] for Experiment 1 was 0.5 (95%CI 0.03 to 0.8), and for Experiment 2 was 0.96 (95%CI 0.92 to 0.98). Results from Experiment 2 suggested more strongly that participants under predicted the number of repetitions they could perform to MF; though could still not wholly exclude an effect within the range of the MDC. The internal meta-analysis echoed the results of Experiment 2 supporting that participants under predicted. These results are mostly in line with previous findings (Hackett et al., 2012, 2016; Giessing et al., 2016a,b; Altoé Lemos et al., 2017; Steele et al., 2017a; Zourdos et al., 2019; Emanuel et al., 2020; Hughes et al., 2020; Mansfield et al., 2020). However, in contrast with prior research this study is the first to examine predictive ability at the sdRM/0RIR. Further, it is the first to use a deception design thus reducing potential demand characteristics from influencing results. This study also offers a behavioral test of the congruence of perception of effort and actual effort in resistance exercise tasks.

Many authors have examined the accuracy of participants’ ability to predict proximity to MF across different exercises using both single and multiple sets, varying relative loads, and predictions both *a priori* and during sets at varying proximities to MF (Hackett et al., 2012, 2016; Altoé Lemos et al., 2017; Steele et al., 2017a; Emanuel et al., 2020; Hughes et al., 2020; Mansfield et al., 2020). The overall results of these studies suggest participants generally under predict the number of repetitions they can perform to MF whether predictions are made *a priori* to initiation of exercise, or at varying degrees of proximity to actual MF. Improved accuracy, which has been shown with subsequent sets (Hackett et al., 2012; Emanuel et al., 2020; Mansfield et al., 2020) or heavier loads (Altoé Lemos et al., 2017; Emanuel et al., 2020; Hughes et al., 2020), would suggest proximity to MF may play a role, though accuracy may still be imperfect. Indeed, Zourdos et al. (2019) found that, despite improved accuracy of predictions with closer proximity to MF, participants still under predicted when they thought they were 5, 3, and 1 repetition away from MF (difference between predicted and actual of 5.15 ± 2.92, 3.65 ± 2.46, and 2.05 ± 1.73 for 5RIR, 3RIR, and 1RIR, respectively). In the current study, participants were instructed to perform a single set to either sdRM (i.e., 0RIR) or MF. Prior studies have not examined this context though it has been speculated that predictive ability would be improved with greater proximity to MF (Mansfield et al., 2020). Furthermore, experienced (>1 year) participants were chosen following prior suggestions that participants predictive ability may improve with training experience (Helms et al., 2016; Steele et al., 2017a). However, our results suggest that even during the gestalt experiences of attempting to get as close as possible, but not reach MF, resistance training experienced participants (>1 year) are still not adequately accurate in their predictions. This is in accordance with other findings in trained participants (Hackett et al., 2012, 2016; Steele et al., 2017a; Zourdos et al., 2019).

Congruence of the perception of effort compared with the actual effort required may play an essential role in individuals’ ability to predict proximity to MF. The actual effort required to complete a task can be defined as a function of the absolute demands of the task and the current ability to meet those demands (Steele, 2020). As such, in resistance training for example, the load can affect the actual effort required (higher loads will require greater actual effort to lift them), as can fatigue (reduced capacity) insidious to continued performance (as a set of repetitions progresses each repetition will require greater and greater effort). Both load and fatigue therefore are related to the actual effort required to complete a resistance exercise task. Indeed, the perception of load (i.e., task demands) as well as fatigue (i.e., capacity) and thus perception of effort (Steele, 2020) might determine the accuracy of predictions of proximity to MF. However, though related, the perception of these three (load, fatigue, and effort) can be differentiated (e.g., Buckingham et al., 2014; Micklewright et al., 2017). Despite this, studies suggest trainees may anchor their perceptions of effort upon other salient perceptions; for example, discomfort (see Steele et al., 2017a). This has been argued to be a potential factor influencing predictive accuracy (Steele et al., 2017c). Although the combined perceptions associated with the gestalt experience of performing a resistance exercise bout (i.e., perceived fatigue, effort, and discomfort) typically intensify with closer proximity to MF, the salience of discomfort may overwhelm and influence prediction. In the current study as well as in previous studies (Hackett et al., 2012, 2016; Giessing et al., 2016a,b; Altoé Lemos et al., 2017; Steele et al., 2017a; Zourdos et al., 2019; Emanuel et al., 2020; Hughes et al., 2020; Mansfield et al., 2020), it might have been the case that participants anchored their perception of effort upon their perceptions of discomfort, leading to an overestimation of effort and thus under prediction of how close they were to MF. As outlined by Steele et al. (2017c), without clear instructions, anchoring of effort based on other perceptions such as discomfort seems to happen during resistance exercise.

Poor predictive ability may have implications for managing resistance training through predictions of proximity to failure; this includes both application of sdRM and RIR scales more generally. It may be the case that an initial period of familiarization with the scale (including with training to MF so as to provide an experiential top anchor under supervised conditions) is required to improve predictive accuracy and the RIR scales utility (Helms et al., 2016). Indeed, where it has been recently applied with strength athletes such as powerlifters, an initial familiarization period has been included (Androulakis-Korakakis et al., 2018). Trainees and coaches should be aware that programming resistance training using RIR might result in systematically training with a lower than intended effort if accuracy in predicting proximity to MF is poor. This may have potential to impact upon their adaptations to resistance training (Giessing et al., 2016a,b). However, a limitation of this study should be acknowledged. We did not ask the participants regarding the specifics of their prior training history and thus the extent to which they trained specifically with the knee extension exercise and to MF are unclear. It is indeed possible that, though participants were ‘trained,’ they may have been relatively inexperienced in the procedures performed in the present experiments (i.e., training to MF). Thus, the generalizability of our findings to ‘trained’ persons should be treated with the appropriate caution.

## Conclusion

In conclusion, our results seem to suggest that trained participants with a minimum of 1-year training experience are not adequately accurate at predicting proximity to MF during the gestalt experience of resistance exercise. Further research should look to identify the information that persons utilize to form their predictions during resistance exercise and other physical tasks (i.e., discomfort, fatigue, effort). The inaccuracy of prediction for even trained persons has implications for the control of effort (i.e., proximity to MF) during resistance training. Whether or not predictive ability is sufficient is still yet to be determined as some research suggests effort is an important variable for determining adaptations to resistance training. However, these results suggest this is something to be aware of and will be an issue for controlling submaximal effort. In fact, it is suspected that people on average are inaccurate at gauging effort during submaximal conditions.

## Data Availability Statement

The datasets presented in this study can be found in online repositories. The names of the repository/repositories and accession number(s) can be found below: https://osf.io/s9yqk/.

## Ethics Statement

The studies involving human participants were reviewed and approved by Health, Exercise, and Sport Science ethics committee at Solent University (ID: standish-hunt2018). The patients/participants provided their written informed consent to participate in this study.

## Author Contributions

JS and HS-H conceived of the study. JS, CA, HS-H, and JF designed the study. JS, CA, HS-H, PA-K, NM, TG, and AH conducted the experiments and collected the data. JS analyzed the data. JS, CA, HS-H, PA-K, NM, TG, AH, JF, PG, and JG all contributed to the interpretation of findings and manuscript preparation. All authors contributed to the article and approved the submitted version.

## Conflict of Interest

The authors declare that the research was conducted in the absence of any commercial or financial relationships that could be construed as a potential conflict of interest.

## Footnotes

1 One of the researchers had moved during the study to a separate location and had access to a knee extension resistance machine. Thus, to contribute further data that might improve meta-analytic estimates (see the section “Statistical Analysis”), the researcher was able to recruit some additional participants and test them.
